# The Ultrahigh Adsorption Capacity and Excellent Photocatalytic Degradation Activity of Mesoporous CuO with Novel Architecture

**DOI:** 10.3390/nano13010142

**Published:** 2022-12-28

**Authors:** Jing Ni, Jianfei Lei, Zhaowu Wang, Lanlan Huang, Hang Zhu, Hai Liu, Fuqiang Hu, Ting Qu, Huiyu Yang, Haiyang Yang, Chunli Gong

**Affiliations:** 1 School of Chemistry and Material Science, Hubei Engineering University, Xiaogan 432000, China; 2 School of Physics and Engineering, Henan University of Science and Technology, Luoyang 471023, China; 3 School of Materials Science and Engineering, Hubei University, Wuhan 430000, China

**Keywords:** mesoporous, photocatalyst, adsorption, methyl orange, CuO

## Abstract

In this paper, mesoporous CuO with a novel architecture was synthesized through a conventional hydrothermal approach followed by a facile sintering procedure. HR-TEM analysis found that mesoporous CuO with an interconnected pore structure has exposed high-energy crystal planes of (002) and (200). Theoretical calculations indicated that the high-energy crystal planes have superior adsorption capacity for H^+^ ions, which is critical for the excellent adsorption and remarkable photocatalytic activity of the anionic dye. The adsorption capacity of CuO to methyl orange (MO) at 0.4 g/L was approximately 30% under adsorption equilibrium conditions. We propose a state-changing mechanism to analyze the synergy and mutual restraint relation among the catalyst CuO, H^+^ ions, dye and H_2_O_2_. According to this mechanism, the degradation rate of MO can be elevated 3.5 times only by regulating the MO ratio in three states.

## 1. Introduction

Efficient and thorough treatment of organic contaminants in wastewater has been a great challenge in the field of environmental protection. For removing dye contaminants in wastewater, one of the green and sustainable technologies is photocatalysis driven by solar energy, in which the photocatalytic reactions occur on the surface of an illuminated semiconductor where photo-generated electrons and holes are formed. Generally, titanium dioxide (TiO_2_) [1,2,3,4,5] and zinc oxide (ZnO) [6,7,8,9,10] have been undoubtedly demonstrated to be the popular photocatalysts for the oxidative decomposition of many organic compounds under UV irradiation; however, unfortunately, their wide band gaps (~3.2 eV) [10,11] limit further applications in the visible regions (λ > 400 nm).

Cupric oxide (CuO) is a narrow band gap semiconductor (1.2–1.7 eV) that can be considered a promising photocatalyst directly excited by visible light [12,13,14,15]. However, such a narrow band gap easily leads to a rapid recombination of photogenerated electrons and holes, hence resulting in short-lived carriers unless oxidants that can be reduced to other radicals by combining photogenerated electrons are added. Therefore, CuO is employed for the photocatalytic decomposition of organic pollutants, usually in the presence of H_2_O_2_ [16,17,18], which is the simplest oxidant that can easily generate hydroxyl radicals (·OH) on self-decomposition over CuO catalysts. Generally, it is commonly agreed that the degradation of organic dyes takes place primarily on and near the catalyst surface [19,20], so we believe that larger surface areas mean better catalytic performance. Based on this opinion, many researchers are focused on developing novel structures and improving the surface areas of the catalysts to further enhance their performance [21,22,23]. In fact, the advanced catalytic activities can mainly result not only from the larger surface areas but also from the more active surface sites of the catalysts [24,25]. 

In this work, we use a thermodecomposition method to fabricate mesoporous CuO architecture similar to our previous work [26], which is a promising approach to produce mesoporous oxides at a relatively low cost. The process includes a traditional hydrothermal method and a facile sintering procedure. Thin blocks of copper nitrate hydroxide (Cu_2_(NO_3_)(OH)_3_) can be prepared via hydrothermal method. The subsequent sintering procedure would yield an interesting result. This process is similar to that of the formation of pores in bread during the baking procedure. The sintering processtakes advantage of the formation and escape of gases during thermal decomposition, forcing the uniform rearrangement of CuO nanocrystals Finally, a hierarchical copper oxide with interconnected pores can be obtained, which demonstrates excellent adsorption capacity and high photocatalystic activity for methyl orange (MO).

Herein, it is more noteworthy that a peculiar dye release phenomenon was observed after the adsorption–desorption equilibrium of MO that has not attracted the attention of other researchers thus far. The simulation results show that the exposed polar surface of the CuO crystal has a large surface energy and excellent adsorption ability for H^+^ ions. We proposed a state-changing mechanism and analyzed the adsorption, desorption and photocatalytic degradation of MO on the CuO surface. The change in the MO state (from State I to State II) caused by the temporary consumption of H^+^ ions is a key factor leading to the noteworthy release of MO molecules from the CuO surface. Based on this mechanism, the degradation rate can be increased 3.5 times by adjusting the proportion of MO in the three states.

## 2. Experimental

### 2.1. Materials

Methyl orange was purchased from Shanghai Aladdin Bio-Chem Technology Co., Ltd. Hydrogen peroxide (30%) was supplied by Tianjin Tianli Chemical Reagent Co., Ltd. (China). Copper nitrate (Cu(NO_3_)_2_·3H_2_O), urea (CO(NH_2_)_2_) and all other reagents were supplied by Sinopharm Chemical Reagent Co., Ltd. (China).

### 2.2. Synthesis of Mesoporous CuO

Mesoporous CuO architecture was synthesized using a conventional hydrothermal approach followed by a facile sintering procedure. First, Cu_2_(NO_3_)(OH)_3_ with perfect crystallinity was obtained via the hydrothermal method. In a typical procedure, 1.208 g Cu(NO_3_)_2_·3H_2_O and 0.300 g CO(NH_2_)_2_ were dissolved in 50 ml of distilled water. After stirring for 10 min, the whole homogeneous mixture was then transferred into a 100 mL Teflon-lined stainless steel autoclave and incubated at 120 °C for 5 hours. After the hydrothermal reaction, the as-formed precipitate was centrifuged, washed with deionized water and dried at 70 °C to obtain green powders as an initial product of Cu_2_(NO_3_)(OH)_3_. Second, initial products of Cu_2_(NO_3_)(OH)_3_ were further calcined in air at 400 °C for 1 hour to obtain mesoporous CuO.

### 2.3. Characterization 

The crystalline structures and morphologies of the as-prepared samples were characterized by using an X-ray diffractometer (XRD, Bruker D8 Advance, Germany) with Cu K_α_ rays (λ = 1.5406 Å) and scanning electron microscopy (SEM, HITACH SU8010, Japan). High-resolution images and electron diffraction patterns of the samples were obtained via transmission electron microscopy (TEM, JEM-2100HR, Japan). The surface area of the mesoporous CuO was measured using Brunauer–Emmett–Teller (BET, TristarⅡ3020, America). 

The optical properties of CuO and the concentration of MO aqueous solution were analyzed using a UV–Vis spectrophotometer (UV2600, Japan). The adsorption of MO molecules on the CuO surface was detected using a Fourier transform infrared spectrophotometer (FT-IR, Nicolet 6700, America).

### 2.4. Computational Details

All calculations were based on density functional theory (DFT), using the Vienna ab initio Simulation Package (VASP) code [27]. The exchange and correlation terms were described using the general gradient approximation (GGA) in the scheme of Perdew–Burke–Ernzerhof (PBE) [28]. Core electrons were described by pseudopotentials generated from the projector augmented wave method [29], and valence electrons were expanded in a plane-wave basis set with an energy cutoff of 490 eV. The Monkhorst–Pack k-points grid [30] of 8×10×8 was used for structure relaxation. Both the positions of all ions and the unit cell parameters were relaxed to minimize the atomic forces and the total energy with a force convergence < 0.01 eV/Å. The calculated equilibrium lattice constants, a = 4.63 Å, b = 3.45 Å, c = 5.11 Å, α = γ= 90°, and β = 99.04°, compared well with the experimental values. The slab model was used to simulate the surfaces of CuO, where the slabs were separated by a vacuum region of 15 Å in the z direction. The adsorption energy was defined as: E_ad_ = E_all_ − E_surface_ − E_H_, where E_all_ and E_surface_ are the energies for adsorption configurations and surfaces, respectively, and E_H_ is the energy of an isolated H atom.

### 2.5. Adsorption Property 

To evaluate the adsorption property of mesoporous CuO, a series of experiments were performed in MO solutions with different pH values. After adding CuO powder (10 mg) to MO solution (25 ml, 10 mg/L) and fully adsorbing in the dark, each adsorption spectra of MO solution was measured.

The adsorption capacity (%) was calculated according to the following formula

(1) $$\mathrm{Adsorption}\;capacity(\%)=\frac{A_{0}-A_{ads}}{A_{0}}\times 100\%$$

where *A*_0_ and *A_ads_* are the initial and saturated absorbances of the MO solution at 464 nm, respectively. 

In particular, for the initial acidic MO solution (pH < 3.1), the position of the maximum absorption peak appears at 505 nm. However, when CuO was added, the maximum absorption peak of the adsorbed saturated MO solution would return to 464 nm due to the strong adsorption ability of CuO on H+ ions. Therefore, in this paper, the absorbance of the neutral solution was chosen as A_0_, which had the same concentration with the initial acidic MO solution.

### 2.6. Photocatalytic Activity

The photocatalytic activity of mesoporous CuO was evaluated through the degradation of MO under a tungsten lamp. In a typical experiment, as-prepared CuO samples (40 mg) were divided into two equal parts, and each sample was dispersed in MO solution (10 mg/L, 50 ml) with initial pH values of 2.5 and 7.0. Before photocatalysis, 1 mL H_2_O_2_ (5%) was added to the MO solution, and then the mixture was stirred by magnetic stirring immediately and exposed to a 200 W tungsten lamp with a light intensity of 10 mW/cm^2^ at the surface of the dye solution. At a given time interval, approximately 4 ml of the mixture was removed and immediately centrifuged. The corresponding absorption spectra were determined using UV–visible absorption spectroscopy.

The absorbance value of 464 nm was selected to calculate the degradation efficiency and removal rate of MO in the solution. The removal rate of MO was calculated as follows: removal rate (%) = (*C*_0_ − *C_t_*)*/C*_0_ × 100%, where *C*_0_ and *C_t_* represent the concentration of MO at the reaction carried out at 0 and t times, respectively. 

## 3. Results and Discussion

### 3.1. Structural Characteristics Analysis

**Figure 1:**
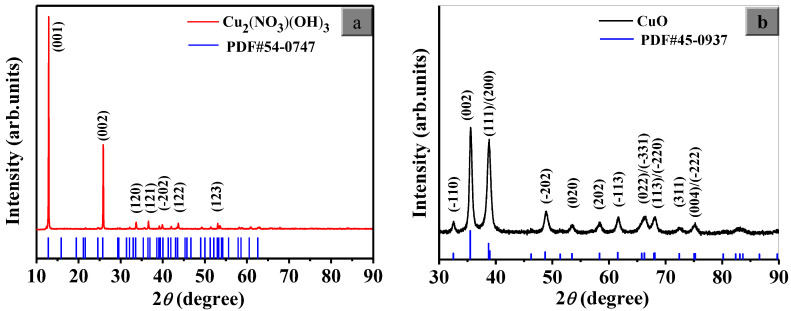
XRD patterns of (**a**) Cu_2_(NO_3_)(OH)_3_ and (**b**) CuO.

Figure 1 shows the XRD patterns of the as-prepared samples. It can be seen from Figure 1a that the sample obtained from the first procedure has fine crystallinity, and all diffraction peaks were good with the monoclinic single crystal Cu_2_(NO_3_)(OH)_3_ (PDF# 54-0747) index. In Figure 1b, the calcined sample has sharp characteristic peaks at 35.6°, 38.7° and 38.9°, which are indexed to the (002), (111) and (200) lattice planes of monoclinic CuO, respectively. No characteristic peaks of Cu_2_(NO_3_)(OH)_3_ are observed, indicating complete conversion to CuO after the secondary calcination procedure as the following reaction.

(2) $$Cu_{2}\left(NO_{3}\right){\left(OH\right)}_{3}\overset{\Delta }{\to }CuO+NO_{2}↑+O_{2}↑+H_{2}O↑$$

**Figure 2:**
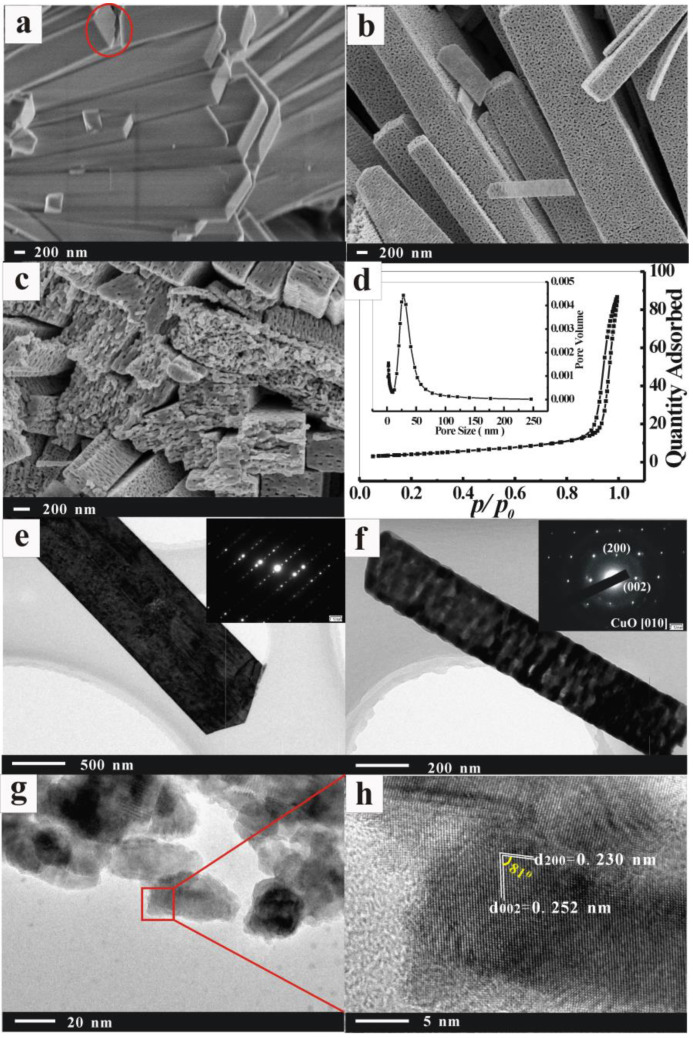
FESEM images of (**a**) Cu_2_(NO_3_)(OH)_3_ and (**b**,**c**) CuO. (**d**) The N_2_ adsorption–desorption isotherm together with the BJH pore size distribution plots (inset) of CuO sample. TEM images and SAED patterns (inset) of (**e**) Cu_2_(NO_3_)(OH)_3_ and (**f**) CuO. (**g**,**h**) HR-TEM images of CuO.

In addition, since it is known from the reaction that heating promotes the decomposition of Cu_2_(NO_3_)(OH)_3_ into NO_2_, O_2_ and H_2_O(g) gasses, we can infer that the following sintering procedure yields interesting results. Because of the formation and the escape of these gasses, which act as templates, the crystalline particles of CuO may be forced to rearrange consistently, and many pores will be left in the internal crystal. This inference can be confirmed by the following SEM images and TEM images shown in Figure 2. Obviously, the surface is very smooth as well as the interior (marked as red circle in Figure 2a) for Cu_2_(NO_3_)(OH)_3_ samples, while a large number of pores are present on the surface of the calcined samples (Figure 2b). After calcination, the morphology of the sample remained largely unchanged except for the formation of pores, which were not only on the surface but also on the inside of the CuO sample (Figure 2c), with an average pore size of approximately 20–30 nm. Figure 2d shows the N2 adsorption–desorption isotherm and the BJH pore size distribution plots (inset) of porous CuO samples, with a BET surface area value of 15.28 m^2^/g and a BJH desorption average pore width of 25.58 nm. The size of 25.58 nm indicates that the material we prepared belongs to the mesoporous structure. The high specific surface area of 15.28 m^2^/g may lead to more surface active sites, which facilitate the adsorption of dye molecules in solution. Therefore, the porous nanostructure of the catalyst may provide more accessible surface areas to increase the active reaction sites, which facilitate their application in catalysts.

Figure 2e–h present the TEM images, selected area electron diffraction (SAED) patterns and high-resolution transmission electron microscopy (HR-TEM) images of the prepared samples. Clearly, typical mesoporous features are observed in sintered samples, while perfect monocrystalline architecture is observed in nonsintered samples. Furthermore, as shown in Figure 2f, the orientation of all crystal particles in the sintered sample is similar, indicating that the arrangement of crystal particles during sintering is consistent. The SAED patterns (insert in Figure 2f) are very consistent with the results in Figure 2f. The HR-TEM image in Figure 2h shows the CuO crystalline lattice fringes with interplanar spacings of 0.252 nm and 0.230 nm, corresponding to the (002) and (200) facets of monocline CuO, respectively. Of particular note is the interfacial angle of 81° indicated in Figure 2h, which is the same as the theoretical value of the angle between (002) and (200) (81.2°), indicating that the as-prepared CuO samples expose (002) and (200) high-energy crystal surfaces. This preferred orientation is of particular interest, as high-energy facets may possess superior adsorption properties.

### 3.2. The Adsorption Capability of Mesoporous CuO

**Figure 3:**
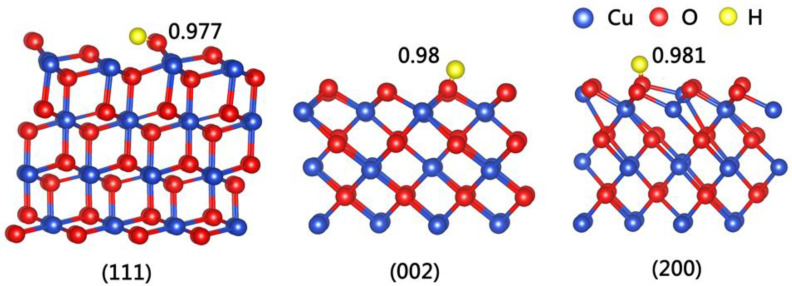
The geometric structures of H atom on various surfaces of CuO crystal.

According to the literature [31,32,33,34], the acidity of the system has a significant effect on the degradation of MO. In other words, H^+^ ions can alter the surface properties of catalysts or participate in degradation reactions [35]. To better understand the role of H^+^ ions during the catalytic process, the adsorption of H^+^ ions on the surface of CuO was studied using first-principles calculations. The calculated results are summarized in Table 1, and the typical calculation models are shown in Figure 3.

It is evident that the exposed polar surfaces of CuO crystals, such as (200) and (002), have high surface energy and superior adsorption capacity to H^+^ ions. We know that MO is a polar molecule that exists in solution in the form of anionic salts. It is generally accepted that the catalytic process is mainly related to the adsorption and desorption of MO molecules on the surface of the catalyst [36,37]. Thus, a high adsorption capacity of MO on the catalyst surface is conducive to improving the efficiency of photocatalytic degradation. Therefore, it can be inferred that the concentration of H^+^ ions on the surface plays an important role in the adsorption of MO and directly determines the adsorption capacity of MO.

**Figure 4:**
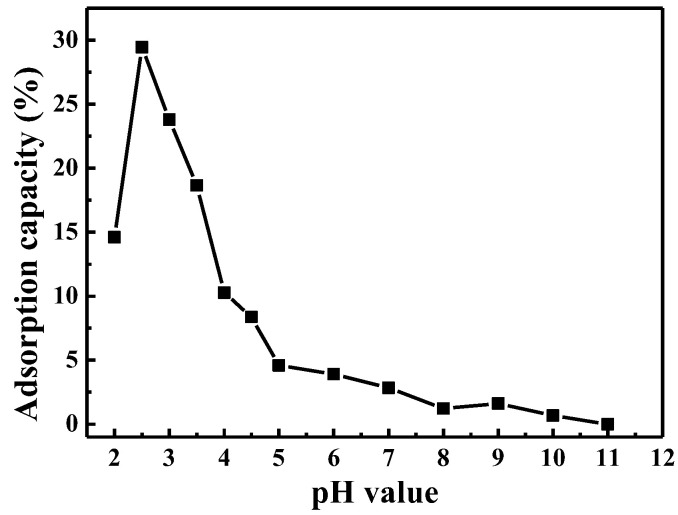
Effect of initial pH value on the adsorption of MO (MO: 10 mg/L, 50 mL; CuO: 20 mg).

To investigate the effect of initial pH on MO adsorption, a series of experiments were performed under the same conditions (50 ml solution containing 20 mg CuO and 10 mg/L MO), in addition to pH value differences (pH = 2–11). The point of zero charge (pH_pzc_) on the surface of CuO is reported [38,39,40] at 7.4–7.8; that is, when the pH value < pH_pzc_, the adsorption point on the surface of CuO is positively charged. This will facilitate the electrostatic adsorption of anionic molecules on the surface of CuO. As shown in Figure 4, the adsorption of MO on the surface of CuO strongly depended on the pH values, and clearly, the acidic solution is a benefit for its adsorption. The adsorption capacity (%) reached a maximum of 29.46% at pH = 2.5 while there were much lower values in basic (pH = 7.0, 2.83%) or alkaline solutions. XRD, TEM and theoretical analysis confirm that the crystal planes (200) and crystal planes (002) with large adsorption energy H^+^ ions are exposed, causing the adsorption of H^+^ on the mesoporous copper oxide surface. 

**Figure 5:**
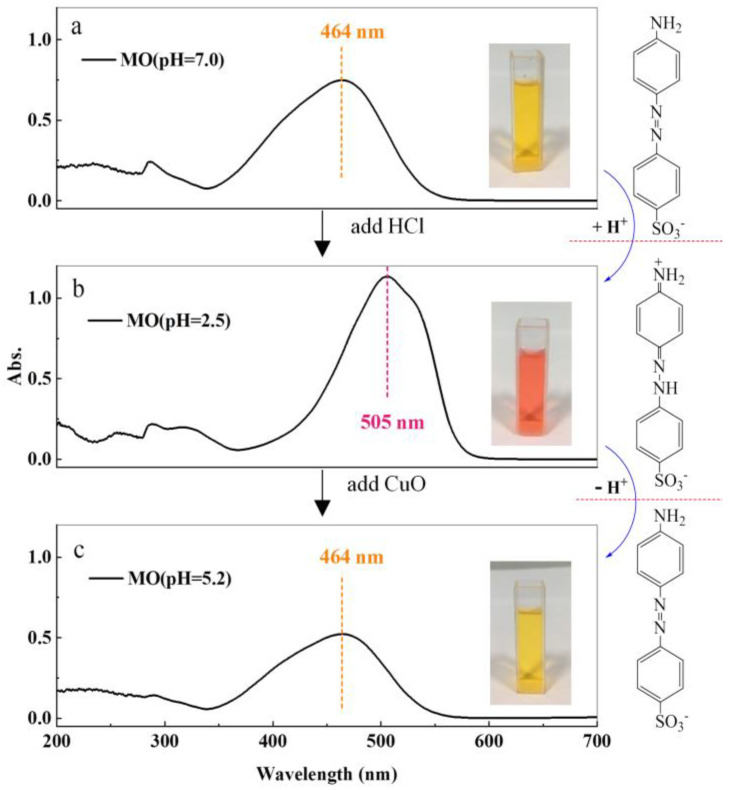
Changes of maximum adsorption peak and color in MO solution and evolution of MO molecular structure under H^+^ ions. (**a**) MO solution with pH = 7.0. (**b**) MO solution with pH = 2.5. (**c**) The results of MO supernatant after H+ adsorption on CuO surface.

In our study, the adsorption behavior of H^+^ ions on the CuO surfaces was also evidenced by experimental phenomena. As shown in Figure 5, the position of the maximum adsorption peaks varies with the color of the solution. It is well known that the structural change of MO can cause color changes in the solution, and the acidity of the solution is one of the major contributors (MO is mainly in the red quinine structure when pH < 3.1 and in the yellow azo structure when pH > 4.4). Clearly, once CuO powders are added to an acidic MO solution (pH = 2.5), the color of the solution changes from red to yellow, and the pH value changes from 2.5 to 5.2 correspondingly. 

Based on the above results, in acidic systems, the surfaces of CuO should be positively charged due to the adsorption of a large amount of H^+^ ions (here named CuO_(H)_ surfaces), which further leads to the electrostatic adsorption of MO (anionic dyes) onto the CuO_(H)_ surfaces. In alkaline conditions, there are fewer H^+^ ions for CuO trapping and tiny amounts of MO are adsorbed on the surface of CuO. In addition, the adsorption capacity of MO increases with decreasing pH and increases dramatically under acidic conditions, as shown in Figure 4. The adsorption of MO on the surface of copper oxide is further illustrated by infrared spectroscopy (Figure S1). Notably, when the pH is below 2.5, the dissolution of CuO must be considered due to the strong acidity of the system. Based on these results, MO solution with pH = 2.5 was selected as the study group, and pH = 7.0 was selected as the comparison group.

### 3.3. The Roles of H^+^ Ions and H_2_O_2_

**Figure 6:**
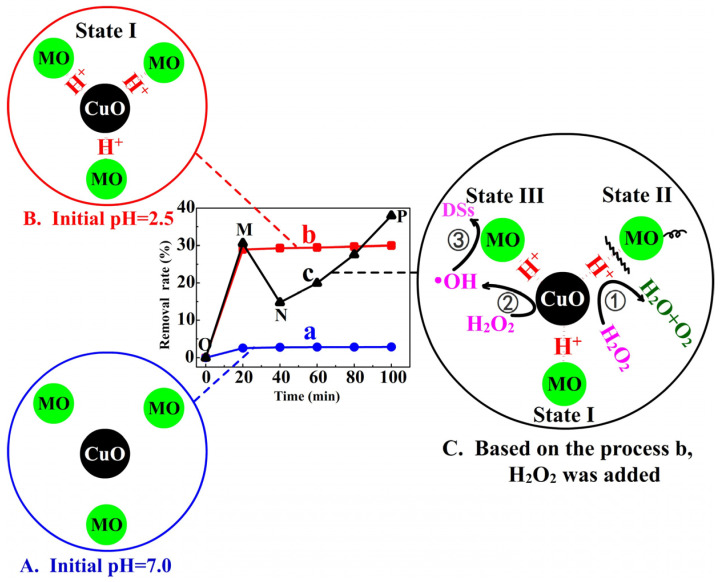
The state-changing schematic diagram of MO molecules on the catalyst surface in MO + CuO + H_2_O_2_ system. (**A**) CuO + neutral MO solution. (**B**) CuO + MO solution (pH = 2.5). (**C**) When CuO + MO solution (pH = 2.5) reached adsorption–desorption equilibrium, H_2_O_2_ was added. (MO: 10 mg/L, 50 mL; CuO: 20 mg; H_2_O_2_: 5%, 1 mL).

The adsorption and degradation of MO on the surface of copper oxide are analyzed in pH = 7.0 and pH = 2.5 systems. As shown in Figure 6, Figure 6A,B refer to the adsorption equilibrium processes of MO on the CuO surfaces in pH = 7.0 and pH = 2.5 systems, respectively. Figure 6C represents the process of adding 5% H_2_O_2_ (1 mL) after CuO + MO solution (pH = 2.5) reaches the adsorption–desorption equilibrium.

In Figure 6A with the solution pH = 7.0, only a small amount of H+ ions can be adsorbed by CuO, and correspondingly, a very small amount of MO molecules were adsorbed on the surface of CuO through electrostatic adsorption. Therefore, the removal rate (%) of MO in the solution of this system was only 2.83% (according to curve a), and the vast majority of MO molecules are distributed in the solution.

In Figure 6B with an initial pH = 2.5, a large number of H^+^ ions were adsorbed on the CuO surface. Subsequently, the anionic MO molecules are well adsorbed by electrostatic attraction. After 20 min, the removal rate (%) reached a saturation value of 29.46% (according to curve b).

However, in Figure 6C, when H_2_O_2_ was added (point M in curve c) to the adsorption-saturated system B, the removal rate (%) dropped rapidly to 14.75% within 20 min after the addition (M→N process in curve c) and then increased gradually (N→P process).

We propose a state-changing mechanism of MO to explain the above experimental results. In system C, three states of MO should be presented simultaneously on or near the CuO surface, and we mark them as State I, State II and State III. Here, State I represents the simple absorbed MO molecules on the CuO surfaces by electrostatic adsorption, and States II and III are the MO molecules that are changed from State I due to the addition of H_2_O_2_. The relevant reactions that occurred on the CuO surfaces during the M→N→P process can be expressed as follows:

reaction ①:
  (3) $$\begin{array}{l} H_{2}O_{2}{}_{\left(CuO\right)}+2H^{+}{}_{\left(CuO\right)}+2e^{-}\to 2H_{2}O\\ H_{2}O_{2}{}_{\left(CuO\right)}-2e^{-}\to 2H^{+}+O_{2} \end{array}\}\begin{array}{c} H_{2}O_{2}{}_{\left(CuO\right)}\to 2H_{2}O+O_{2} \end{array}$$
reaction ②
  (4) $$H_{2}O_{2}{}_{\left(CuO\right)}\to \cdot OH$$
reaction ③
  (5) $$MO_{\left(CuO\right)}+\cdot OH\to \mathrm{degradation}\;\mathrm{species}\;(DSs)$$

where the subscript CuO represents the corresponding component adsorbed on the CuO surface. According to Equation (3), H^+^_(CuO)_ ions are despoiled by H_2_O_2(CuO)_ molecules from the surfaces of CuO resulting in a decrease in the H^+^_(CuO)_ concentration, which leads to the vast release of MO from the CuO surfaces due to the weakening of electrostatic adsorption to form desorbed MO (State II). Although H^+^ ions are reproduced in reaction ① in Figure 6C, the process of MO readsorption to State I is slower than that of MO release. Meanwhile, plenty of gasses can be observed as soon as H_2_O_2_ is added to the system, and we believe that H_2_O_2_ molecules are decomposed with the help of H^+^_(CuO)_ ions and Cu^2+^/Cu^+^ [41,42]. The catalytic decomposition of H_2_O_2(CuO)_ can generate ·OH (or ·OOH and ·O_2_^−^ ) radicals [43,44,45,46] (Equation (4), Fenton-like reaction), which can further degrade MO_(CuO)_ molecules into degradation species (DSs) (Equation (5)). Here, we define these degraded MO_(CuO)_ molecules as State III. 

In our study, as shown in curve c in Figure 6, MO_(CuO)_ molecules are presented only as State I during the O→M process, while the addition of H_2_O_2_ changes this situation, and they are presented as three kinds of states. For the M→N process of curve c, the existence states of MO are dominated mainly by State II, which leads to a declining removal rate (%), and the adsorption (State I) and release (State II) of MO_(CuO)_ reach equilibrium again at the N point. The degradation of MO_(CuO)_ molecules acting as State III mainly occurs in the following process (N→P).

Clearly, H^+^ ions play a key role in the adsorption/desorption of MO. The emergence of MO_(CuO)_ molecules as State I achieved with H^+^ ions is necessary for the following degradation reaction. In essence, however, MO_(CuO)_ molecules as State III are the targets for effective degradation in the presence of the H_2_O_2_ system. Concurrently, the addition of H_2_O_2_ will also consume H^+^_(CuO)_ ions, causing the release of MO_(CuO)_, that is, resulting in the appearance of MO molecules as State II. The locations of the degradation reaction (reactions ② and ③ in Figure 6C) are the effective adsorption positions of mesoporous CuO. 

### 3.4. The Optical Properties of CuO

**Figure 7:**
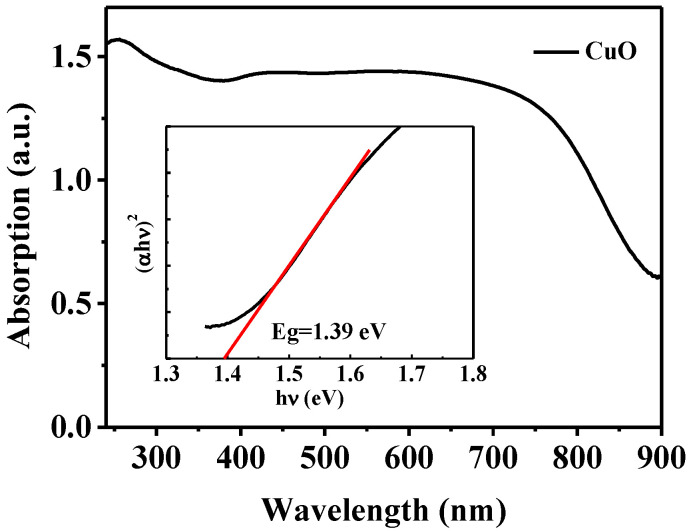
The UV–Vis absorption spectra and Tauc plot of mesoporous CuO.

To confirm the absorption of CuO in the visible range, the obtained samples were tested using UV diffuse reflectance spectroscopy. Figure 7 shows the UV–Vis absorption spectra of mesoporous CuO. It can be seen from the results that CuO has good absorption in the visible region. The optical band gap of mesoporous copper oxide is estimated to be 1.39 eV by extrapolation from the linear part of the Tauc curve. 

### 3.5. Photocatalytic Degradation of MO

As illuminated above, H^+^ ions play a key role in the adsorption process. High adsorption of MO on the CuO surfaces is conducive to improving the efficiency of photocatalytic degradation. Additionally, the addition time of H_2_O_2_ is important, which will cause the state changing of MO molecules on the CuO surfaces.

To test the roles of H^+^ and H_2_O_2_, we designed two series of catalytic degradation experiments with two different ways of H_2_O_2_ addition. The first series is the orderly addition of CuO and H_2_O_2_ in the pH = 2.5 and pH = 7.0 systems, in which H_2_O_2_ is added after the saturated adsorption of MO on the CuO surfaces, and the second series is that CuO and H_2_O_2_ are added simultaneously in the pH = 2.5 and pH = 7.0 systems under light irradiation. 

**Figure 8:**
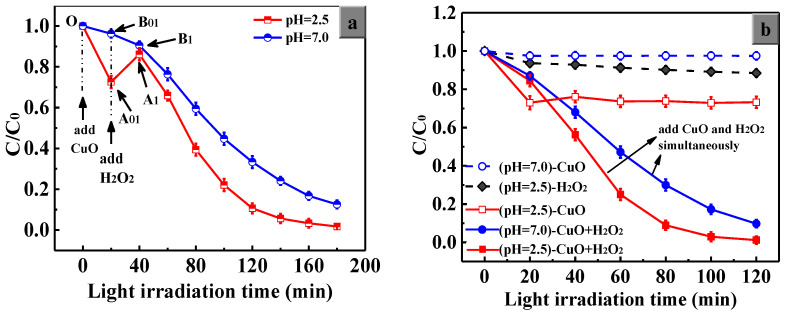
The catalytic performance of the porous CuO powders in the degradation of MO with different initial pH values: CuO and H_2_O_2_ were added (**a**) orderly, (**b**) simultaneously under light irradiation.

Figure 8a shows that the *C*/*C*_0_ decreased to a value of 0.72 in the first 20 min when only CuO was added to the pH = 2.5 system. However, it increased noticeably and rapidly with the addition of H_2_O_2_ due to State II formation. During the second 20 min ($A_{01}\to A_{1}$), the quantity of State I decreased and that of State II increased gradually, and they reached an equilibrium state at the A_1_ point. Thereafter, the *C*/*C*_0_ decreased gradually owing to the main degradation of State III. In the pH = 7.0 system, *C*/*C*_0_ decreased slightly during the initial 40 min ($O\to B_{1}$), which is in deep contrast with that of the pH = 2.5 system. This is because the concentration of H^+^ ions is too low in the pH = 7.0 system, which results in a very small proportion of the generation of State II. Therefore, State III plays a direct role in the reduction of *C*/*C*_0_ during the $O\to B_{1}$ process for the pH = 7.0 system. 

Figure 8b shows the results of the simultaneous addition of CuO and H_2_O_2_ under light. When CuO or H_2_O_2_ was added alone, the system reached adsorption saturation or indicated a low degradation efficiency. However, when CuO and H_2_O_2_ are added at the same time, there is a synergistic effect between them, and finally the MO in the solution can be degraded continuously and rapidly. Since the release and readsorption process of MO molecules did not occur in Figure 8b, the *C*/*C*_0_ value decreased quickly over time. 

**Figure 9:**
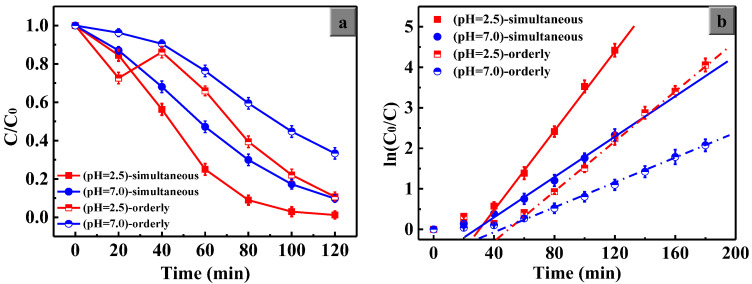
The comparison of catalytic behaviors for CuO under light irradiation with different ways of adding H_2_O_2_. (**a**) C/C_0_ of MO vs. time; (**b**) first-order kinetic plot of ln(C_0_/C) vs. time.

The kinetics of MO degradation in the presence of CuO and H_2_O_2_ under the same ambient conditions were studied. To better analyze the difference between the two ways of adding H_2_O_2_, the results of the two experiments and their first-order kinetics in the degradation of MO with different pH values were compared, as shown in Figure 9. The kinetic expression can be presented as follows [47]:

(6) $$\ln\left(\frac{C_{0}}{C}\right)=k_{app}t$$

where *C*_0_ is the initial concentration of the MO and *C_t_* is the concentration at a given reaction time. 

The apparent first-order rate constant, $k_{app}=Kk$ can be determined from Langmuir–Hinshelwood kinetics at low initial dye concentrations, where *K* is the adsorption equilibrium constant of the dye and its intermediates and *k* is the reaction rate constant. In the linear fitting, one thing to note is that the initial data points are not considered within the data range of the linear fitting, because their adsorption states have not reached adsorption equilibrium. The plot shows an excellent linear correlation, suggesting that the degradation reaction of MO follows first-order kinetics. The parameters of the linear fitting are given in Table 2.

Based on our state-changing mechanism of MO and the results shown in Figure 8 and Figure 9, the adsorption and degradation of MO on the CuO catalyst with the help of H^+^ ions and H_2_O_2_ can be understood as follows:

#### 3.5.1. The Synergistic Effect of H_2_O_2_ and CuO

Figure 8b indicates that few MO molecules were degraded when only H_2_O_2_ was added, and a simple adsorption process of MO can occur on the CuO surfaces (State I) when only CuO powders are added. MO molecules can be degraded efficiently only when H_2_O_2_ and CuO powders are both present in the system. Some research [48,49,50] found that the acidic system had an obvious advantage for enhancing the degradation efficiency. However, there are few detailed explanations for this synergistic effect of H_2_O_2_ and CuO in the acidic system. In our study, we propose the state-changing mechanism to explain this phenomenon well.

#### 3.5.2. The Positive Effect of H^+^ Ions on the Catalytic Behavior of CuO

Clearly, the degradation efficiency in the pH = 2.5 system is significantly superior to that in the pH = 7.0 system due to the violent adsorption of H^+^ ions on the CuO surface, which promotes the effective adsorption of MO molecules on the CuO surface by electrostatic interactions. The high effective adsorption means more active surface sites of porous CuO, which contribute to the degradation of the MO molecules. Additionally, during the $A_{01}\to A_{1}$ process shown in Figure 8a, the added H_2_O_2_ despoils H^+^ ions on the CuO surface, causing the release of MO molecules (State II), which results in the disturbance of the electrostatic adsorption equilibrium between MO and CuO. It takes at least 20 min to achieve a new adsorption equilibrium of MO molecules between State I and State II.

#### 3.5.3. The Advantage of Simultaneous Addition of CuO and H_2_O_2_

In the pH = 2.5 system, it is obvious that the two ways of adding H_2_O_2_ influence the catalytic behaviors of CuO. It can be seen from Figure 8a,b that it took 180 min for the degradation efficiency to exceed 98% for orderly addition (Figure 8a) and only 120 min for simultaneous addition (Figure 8b). Similar results can be observed in the pH = 7.0 system. 

The apparent rate constant can also reflect the catalytic performance of materials directly. As shown in Figure 9b and Table 2, in the pH = 2.5 system, the *k_app_* value is 4.9 × 10^−2^ under the simultaneous addition condition; however, it is only 3.0 × 10^−2^ under the orderly addition condition. For the pH = 7.0 system, the *k_app_* values are 2.4 × 10^−2^ and 1.4 × 10^−2^, respectively. All the data indicate that the simultaneous addition of H_2_O_2_ and CuO into the MO acidic solution is helpful for photocatalytic degradation. The data show that the reaction rate of degradation can be improved by approximately 1.7 times only by changing the ways of adding H_2_O_2_.

Comparing the results of group (pH = 2.5)-simultaneous and group (pH = 7.0)-orderly, the *k_app_* value is elevated 3.5 times only by regulating the proportion of MO in the three states. Based on our state-changing mechanism, we believe that the different proportions of the three states of MO are the main reasons for the difference in the degradation process. 

In the case of orderly addition in acidic solution, based on Equation (3), a large amount of H_2_O_2_ is consumed due to the mass adsorption of MO before H_2_O_2_ is added, thus decreasing the generation rate of State III with the decreased amount of H_2_O_2_. However, the simultaneous addition may effectively improve the situations of State I and State II, which can greatly improve the catalytic activity of CuO.

### 3.6. The Stability and Reusability of CuO

**Figure 10:**
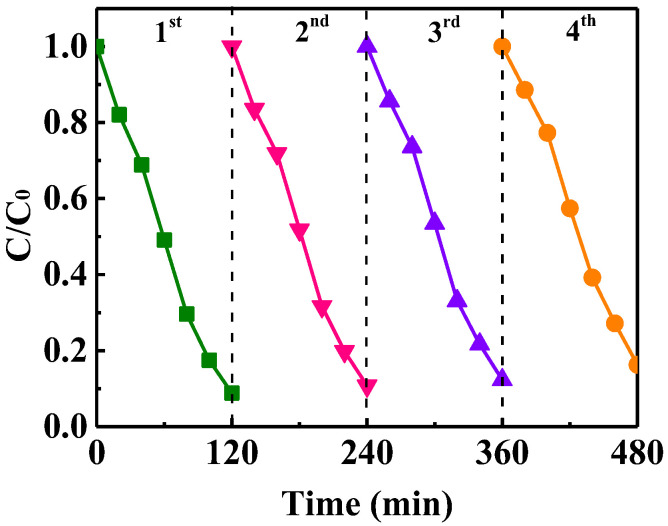
Consecutive runs of the catalytic activity of mesoporous CuO for MO degradation.

In addition to high catalytic activity, the catalyst must also have excellent stability and reusability in successive applications. Mesoporous CuO was investigated for MO degradation by performing recycling experiments under the same conditions. As shown in Figure 10, only a slight decrease in the catalytic activity is observed after 4 consecutive cycles, which illustrates the high durability of the catalyst under visible light. 

### 3.7. Photocatalytic Mechanism

The band structure and band potentials of the photocatalyst have a great influence on photocatalytic performance. The positions of the conduction band (*E_CB_*) and valence band (*E_VB_*) are estimated using empirical formulas [51]:

(7) $$E_{VB}=\chi -E_{e}+0.5E_{g}$$

(8) $$E_{CB}=E_{VB}-E_{g}$$

where *χ* is the geometric mean of the absolute electronegativity of the constituent atoms. For CuO, *χ* is 5.812 eV. *E_e_* (approximately 4.5 eV) is the energy of free electrons on the hydrogen scale. *E_g_* is the energy band-gap, which was estimated to be 1.39 eV according to the UV–Vis spectra. Therefore, *E_CB_* and *E_VB_* are 0.62 and 2.01 (eV vs. NHE) for CuO, respectively.

**Figure 11:**
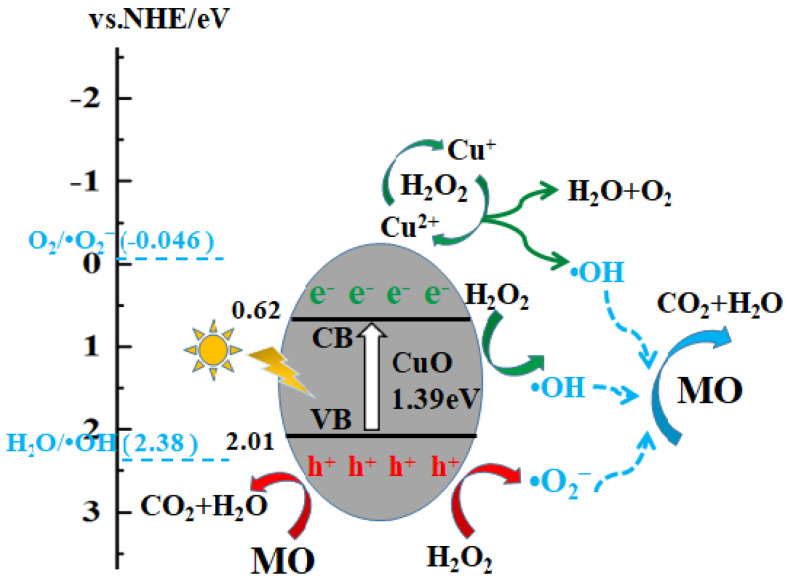
Band-gap structure of CuO and photocatalytic mechanism.

Based on the above analysis, the band-gap structure of CuO and a photocatalytic mechanism are illustrated in Figure 11. Under light irradiation, the photogenerated electrons can be excited from the VB to the CB, and the holes with strong oxidation remain in the VB. The MO molecules adsorbed on the surface of CuO would be oxidized into CO_2_ and H_2_O by the holes in the VB. H_2_O_2_ could also be oxidized to ·O_2_^−^. On the other hand, H_2_O_2_ can trap electrons in the CB and ·OH would be formed. In addition, the redox chain reaction between Cu^2+^/Cu^+^ and H_2_O_2_ can produce H_2_O, O_2_ and ·OH. Since the CB position of CuO is 0.62 eV, which is lower than that of O_2_/·O_2_^−^ (−0.046 eV vs. NHE), photo-generated electrons on the CB cannot reduce O_2_ to ·O_2_^−^. Finally, OH and ·O_2_^−^ produced from H_2_O_2_ and photogenerated holes in the VB can degrade MO molecules into CO_2_ and H_2_O.

## 4. Conclusions

In our current work, mesoporous CuO with novel architecture was obtained using a conventional hydrothermal approach followed by a facile sintering procedure. Mesoporous CuO has a strong adsorption capacity for methyl orange under acidic conditions. We propose a state-changing mechanism to analyze the adsorption/desorption of MO on the CuO surface under the action of H^+^ ions and H_2_O_2_. The reaction rate of degradation was elevated 3.5 times by regulating the proportion of MO in the three states. In addition, degradation experiments show that mesoporous CuO exhibits excellent photocatalytic activity and high durability under visible light. 

## Figures and Tables

**Table 1:** The adsorption energy obtained using the first-principles calculation.

Planes	O–H (nm)	Eads (eV)
(111)	0.977	−4.31
(101)	0.980	−4.53
(011)	0.974	−4.52
(002)	0.980	−5.40
(200)	0.981	−5.49

**Table 2:** The parameters of the linear fitting in Figure 9b.

Case	*k_app_* (×10^−2^)	Intercept	*R* ^2^
(pH = 2.5)-orderly	3.0	−1.49	0.998
(pH = 7.0)-orderly	1.4	−0.57	0.997
(pH = 2.5)-simultanous	4.9	−1.47	0.996
(pH = 7.0)-simultanous	2.4	−0.67	0.989

## Data Availability

Data sharing is not applicable to this article.

## Supplementary Materials

The following supporting information can be downloaded at: https://www.mdpi.com/article/10.3390/nano13010142/s1, Figure S1: (a) Comparison of infrared spectra of CuO before and after MO adsorption. (b) and (c) Local enlarged views of the blue and red dotted areas in Figure (a). “fresh CuO” represents the original CuO sample. “CuO-MO”represents CuO powder adsorbed a certain amount of MO molecules. “used CuO”represents the CuO powder after the complete photocatalytic degradation of surface MO.
