# Supplementary material for: The Ultrahigh Adsorption Capacity and Excellent Photocatalytic Degradation Activity of Mesoporous CuO with Novel Architecture
Source: Nanomaterials (Basel). 2022 Dec 28;13(1):142. doi: 10.3390/nano13010142 (PMC9824582; doi:10.3390/nano13010142)
Supplement: Supplementary file 1 [file nanomaterials-13-00142-s001.zip › nanomaterials-2080067-supplementary.pdf]

Supporting Information

The Ultrahigh Adsorption Capacity and Excellent Photocatalytic Degradation Activity of Mesoporous CuO with Novel Architecture

Jing Ni [1,*], Jianfei Lei [2], Zhaowu Wang [2], Lanlan Huang [1,3], Hang Zhu [1], Hai Liu [1], Fuqiang Hu [1], Ting Qu [1], Huiyu Yang [1], Haiyang Yang [1] and Chunli Gong [1,*]

[1] School of Chemistry and Material Science, Hubei Engineering University, Xiaogan 432000, China; 17756441728@163.com (L.H.); hangzhu5193@163.com (H.Z.); liuhai_218@163.com (H.L.); hufuqiang0301@163.com (F.H.); quting_2020@hbeu.edu.cn (T.Q.); hy-yang_wtu@hotmail.com (Huiyu Yang); yanghaiyang@hbeu.edu.cn (Haiyang Yang)

[2] School of Physics and Engineering, Henan University of Science and Technology, Luoyang 471023, China; leijianfei9966@163.com (J.L.); wangzhaowu@haust.edu.cn (Z.W.)

[3] School of Materials Science and Engineering, Hubei University, Wuhan 430000, China.

* Correspondence: jingni@hbeu.edu.cn (J.N.); chunli.gong@hbeu.edu.cn (C.G.); Tel: +86 0712 2345464(J.N.)

Figures

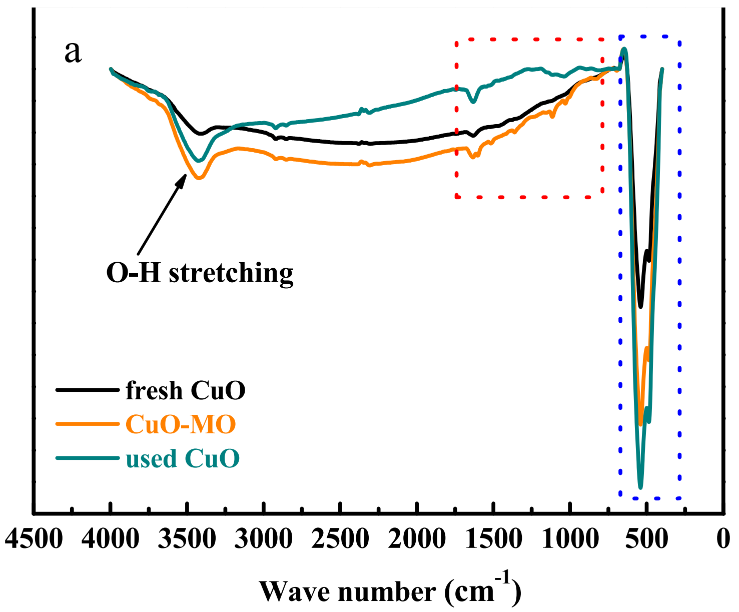

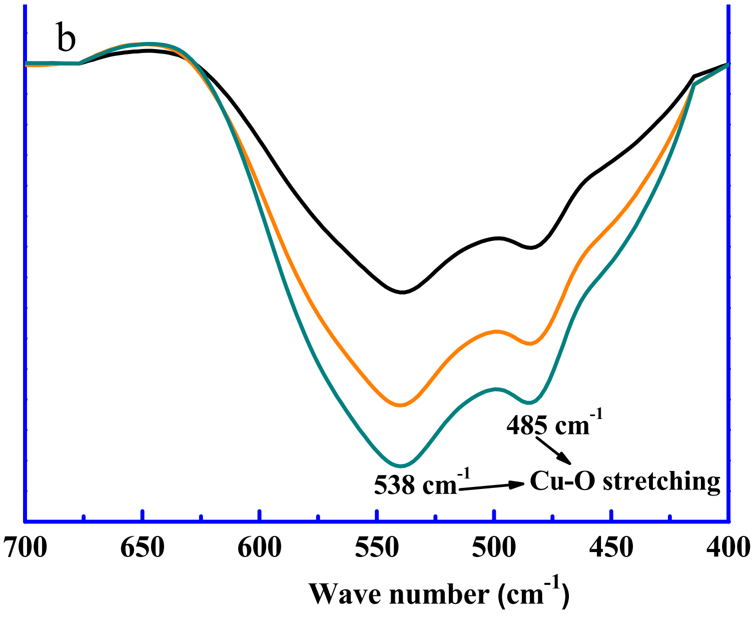

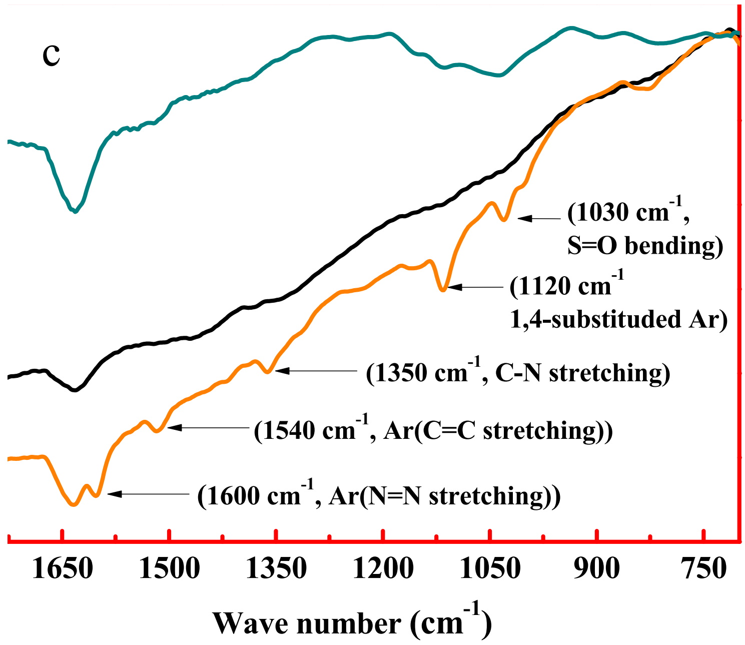

Figure. S1 (a) Comparison of infrared spectra of CuO before and after MO adsorption. (b) and (c) Local enlarged views of the blue and red dotted areas in Figure (a).

"fresh CuO" represents the original CuO sample. "CuO-MO"represents CuO powder adsorbed a certain amount of MO molecules. "used CuO"represents CuO powder after the complete photocatalytic degradation of surface MO.
